# Supporting patients with adherence to glaucoma medication in Ghana

**Published:** 2023-05-22

**Authors:** Boateng Wiafe

**Affiliations:** Technical Advisor: Operation Eyesight Universal, Calgary, Canada.

**Patients need help in many areas – including advocacy for affordable eye medicines.**

**Figure:**
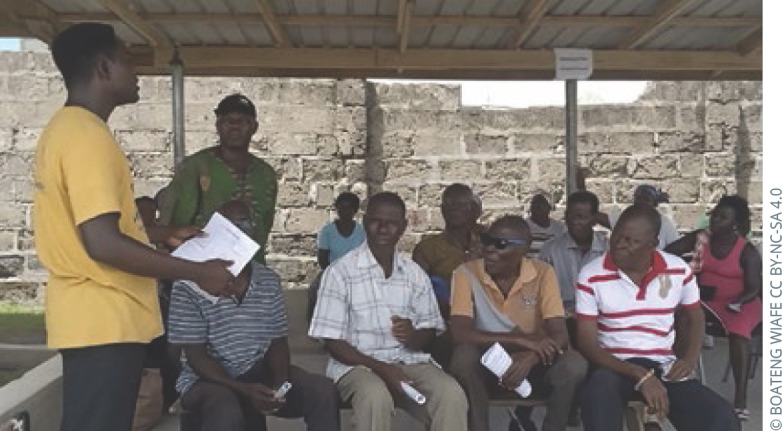
Meeting of a chapter of the Glaucoma Patients Association of Ghana with a guest facilitator. ghana

In Ghana, as in many other low- or middle-income countries, patients who are prescribed medication for glaucoma often stop using their medicines and only come back when their sight has worsened considerably. It is important, from the beginning, to educate patients about the chronic nature of glaucoma and for them to understand that any treatment is lifelong.

Here are some of the reasons patients have given:

- “I used the eye drops only when I felt the symptoms.”
- “I felt better with eye drop treatment. I did not feel the need to use the eye drops all the time, so I use them sometimes.”
- “The frequency of use instructed by the ophthalmologist or pharmacist is too much.”
- “The price of eye drops is too high, so I do not buy it all the time because my pension is very small.”

It is important, from the beginning, to educate patients about the chronic nature of the disease (glaucoma) and for them to understand that any treatment is lifelong. Some patients view the disease to be like malaria, where a stated dose of drug is taken and is sufficient for a cure. They need to understand that this is not the case for glaucoma.

The first and most important step in supporting adherence is therefore patient education and counselling about the importance of – and reasons for – adherence. High patient loads in many eye clinics don't allow specialist eye health care providers to spend much time with the patient, so talking to patients about medication and adherence is often done by nurses, allied health personnel, or trained community eye health volunteers.

In Ghana, patients are counselled by community eye health volunteers, who are trained according to the WHO Afro Primary Eye Care training manual (www.afro.who.int/publications/primary-eye-care-training-manual), which has a strong emphasis on health education and counselling, including counselling patients on using their eye medication.

Other strategies we use to support adherence include:

**Involving family members or care givers.** Making sure that family members or care givers understand what the patient must do, and why, really helps to improve adherence to eye medications, particularly those for long-term conditions such as glaucoma. We always advise elderly patients to come with a care giver if possible. The caregiver is also taught how to apply or instil the medication.

**Phone calls.** We keep a register of all our glaucoma patients, and we call them within 2–3 months to ask how things are going with the medication. We also try to help solve any problems they have.

**Counselling and continuous health education in the community.** Peer counselling, via patient groups, is another means to support patients. Clinicians can encourage patients to form associations and share personal experiences with each other. In Ghana, for example, there is an association of patients with glaucoma called the Ghana Glaucoma Patients Association. The GGPA provides education for new patients, invites counsellors to meetings to address patient concerns, carry out advocacy, and participate in World Glaucoma Week activities to create awareness of glaucoma.

**Advocacy for reduction in medication costs.** Patients can't be expected to be adherent with medication if the medication is not affordable. In countries with health insurance schemes, it is important to talk to insurance providers and make sure that effective eye medication is included on the list of medicines they cover, or on the national approved list. Another option is to ask for donations from pharmaceutical companies and non-governmental organisations on behalf of those who really need medication but cannot afford to pay for it. Operation Eyesight Universal has set aside funds to support some patients in Ghana who are unable to afford their medication, but this is only a short-term solution. Advocacy is key, and one of the things we are working on right now is advocacy to persuade Ghana's national health insurance agency to add more glaucoma medications to their list.

